# Supplementary material for: Ethics practices associated with reusing health data: an assessment of patient registries
Source: BMC Med. 2024 Dec 4;22:577. doi: 10.1186/s12916-024-03799-w (PMC11619252; doi:10.1186/s12916-024-03799-w)
Supplement: Supplementary file 1 — Supplementary material 1. [file 12916_2024_3799_MOESM1_ESM.docx]

**Supplementary materials belonging to the manuscript “Ethics practices associated with reusing health data: An assessment of patient registries”**

Table S1. Patient registries that were assessed in the study.

| **ID** | **Registry Name** | **Geographical origin** | **Last Updated** |
| --- | --- | --- | --- |
| 1 | [ALS Register - Amyotrophic Lateral Sclerosis](https://www.encepp.eu/encepp/viewResource.htm?id=25466) | Germany | 5-9-2018 |
| 2 | [ARS](https://www.encepp.eu/encepp/viewResource.htm?id=24417) | Italy | 13-6-2018 |
| 3 | [BIFAP](https://www.encepp.eu/encepp/viewResource.htm?id=21501) | Spain | 2-11-2017 |
| 4 | [BSRBR - Rheumatic and Musculoskeletal conditions](https://www.encepp.eu/encepp/viewResource.htm?id=23914) | United Kingdom | 8-5-2018 |
| 5 | [BioReg-Austria](https://www.encepp.eu/encepp/viewResource.htm?id=4913) | Austria | 4-10-2013 |
| 6 | [CPRD](https://www.encepp.eu/encepp/viewResource.htm?id=30008) | United Kingdom | 5-6-2019 |
| 7 | [Calliope](https://www.encepp.eu/encepp/viewResource.htm?id=3962) | France | 16-5-2013 |
| 8 | [Caserta database](https://www.encepp.eu/encepp/viewResource.htm?id=45094) | Italy | 11-1-2022 |
| 9 | [DA Germany](https://www.encepp.eu/encepp/viewResource.htm?id=104282) | Germany | 11-4-2023 |
| 10 | [DHR](https://www.encepp.eu/encepp/viewResource.htm?id=24838) | Germany | 16-7-2018 |
| 11 | [DKCC - Skin Cancer](https://www.encepp.eu/encepp/viewResource.htm?id=45254) | Netherlands | 19-1-2022 |
| 12 | [Danish Registries (access/analysis)](https://www.encepp.eu/encepp/viewResource.htm?id=42187) | Denmark | 20-7-2021 |
| 13 | [Drugs and Pregnancy Finland](https://www.encepp.eu/encepp/viewResource.htm?id=5073) | Finland | 1-11-2013 |
| 14 | [ECARUCA - Chromosomal aberrations](https://www.encepp.eu/encepp/viewResource.htm?id=20393) | Netherlands | 7-8-2017 |
| 15 | [EFEMERIS](https://www.encepp.eu/encepp/viewResource.htm?id=104024) | France | 20-3-2023 |
| 16 | [EHR - Blood disorder](https://www.encepp.eu/encepp/viewResource.htm?id=19105) | United Kingdom | 31-5-2017 |
| 17 | [EUGINDAT-PIADATABASE - Primary inherited aminoacid](https://www.encepp.eu/encepp/viewResource.htm?id=19421) | Spain | 2-6-2017 |
| 18 | [EUMDS - Myelodysplastic Syndrome](https://www.encepp.eu/encepp/viewResource.htm?id=19401) | Italy | 1-6-2017 |
| 19 | [EUROPAC - Pancreatic diseases and cancer](https://www.encepp.eu/encepp/viewResource.htm?id=20008) | United Kingdom | 25-7-2017 |
| 20 | [EpiChron Cohort](https://www.encepp.eu/encepp/viewResource.htm?id=22494) | Spain | 6-2-2018 |
| 21 | [Euro WABB - rare genetic disorders](https://www.encepp.eu/encepp/viewResource.htm?id=20472) | United Kingdom | 9-8-2017 |
| 22 | [European Prader-Willi syndrome database](https://www.encepp.eu/encepp/viewResource.htm?id=20063) | United Kingdom | 26-7-2017 |
| 23 | [FTLD Register - Frontotemporal lobar degeneration](https://www.encepp.eu/encepp/viewResource.htm?id=25470) | Germany | 5-9-2018 |
| 24 | [FranceCoag - Haemophilia](https://www.encepp.eu/encepp/viewResource.htm?id=24427) | France | 21-6-2018 |
| 25 | [German CF Registry](https://www.encepp.eu/encepp/viewResource.htm?id=103547) | Germany | 15-2-2023 |
| 26 | [German MS-Register](https://www.encepp.eu/encepp/viewResource.htm?id=47729) | Germany | 15-6-2022 |
| 27 | [HUE-MAN Project - Alpha mannosidosis](https://www.encepp.eu/encepp/viewResource.htm?id=20100) | Norway | 1-8-2017 |
| 28 | [Health Search/CSD LPD](https://www.encepp.eu/encepp/viewResource.htm?id=6874) | Italy | 25-6-2014 |
| 29 | [HemoNED - Haemophilia](https://www.encepp.eu/encepp/viewResource.htm?id=48361) | Netherlands | 26-7-2022 |
| 30 | [Hepather - Hepatitis B and C](https://www.encepp.eu/encepp/viewResource.htm?id=24642) | France | 5-7-2018 |
| 31 | [IMASIS](https://www.encepp.eu/encepp/viewResource.htm?id=104316) | Spain | 4-4-2023 |
| 32 | [IMS - Hospital Treatment Insights - UK](https://www.encepp.eu/encepp/viewResource.htm?id=4824) | United Kingdom | 24-9-2013 |
| 33 | [IMS LifeLink: Hospital Disease Database - Belgium](https://www.encepp.eu/encepp/viewResource.htm?id=4828) | Belgium | 24-9-2013 |
| 34 | [INBC - Blood disorders](https://www.encepp.eu/encepp/viewResource.htm?id=31504) | Italy | 24-9-2019 |
| 35 | [IPCI](https://www.encepp.eu/encepp/viewResource.htm?id=42618) | Netherlands | 26-8-2021 |
| 36 | [Icelandic National Registries](https://www.encepp.eu/encepp/viewResource.htm?id=20569) | Iceland | 16-8-2017 |
| 37 | [LifeLink EMR FR](https://www.encepp.eu/encepp/viewResource.htm?id=2255) | France | 25-11-2011 |
| 38 | [NCRI - Oncology](https://www.encepp.eu/encepp/viewResource.htm?id=25473) | Ireland | 5-9-2018 |
| 39 | [NCRI_PCRS database](https://www.encepp.eu/encepp/viewResource.htm?id=5675) | Republic of Ireland | 27-1-2014 |
| 40 | [NFZ](https://www.encepp.eu/encepp/viewResource.htm?id=20553) | Poland | 17-8-2017 |
| 41 | [Norwegian Porphyria Registry](https://www.encepp.eu/encepp/viewResource.htm?id=47555) | Norway | 2-6-2022 |
| 42 | [Norwegian wholesaler-based drug statistics](https://www.encepp.eu/encepp/viewResource.htm?id=32234) | Norway | 8-11-2019 |
| 43 | [Optimum Patient Care Research Database](https://www.encepp.eu/encepp/viewResource.htm?id=11192) | United Kingdom | 3-10-2015 |
| 44 | [PHARMO Data Network](https://www.encepp.eu/encepp/viewResource.htm?id=50327) | Netherlands | 4-1-2023 |
| 45 | [PROGNOSIS](https://www.encepp.eu/encepp/viewResource.htm?id=23267) | European Union | 21-3-2018 |
| 46 | [Pedianet](https://www.encepp.eu/encepp/viewResource.htm?id=20131) | Italy | 31-7-2017 |
| 47 | [QResearch](https://www.encepp.eu/encepp/viewResource.htm?id=20560) | United Kingdom | 15-8-2017 |
| 48 | [RABBIT](https://www.encepp.eu/encepp/viewResource.htm?id=103477) | Germany | 9-2-2023 |
| 49 | [REHem-AR-SEHOP](https://www.encepp.eu/encepp/viewResource.htm?id=44336) | Spain | 22-11-2021 |
| 50 | [ROR Centro - Oncology](https://www.encepp.eu/encepp/viewResource.htm?id=21283) | Portugal | 19-10-2017 |
| 51 | [ROR Sul - Oncology](https://www.encepp.eu/encepp/viewResource.htm?id=44212) | Portugal | 10-11-2021 |
| 52 | [RORENO - Oncology](https://www.encepp.eu/encepp/viewResource.htm?id=26460) | Portugal | 7-11-2018 |
| 53 | [Real Life Data - Big-Pac](https://www.encepp.eu/encepp/viewResource.htm?id=29236) | Spain | 3-4-2019 |
| 54 | [Reuma.pt - Rheumatic diseases Portuguese Registry](https://www.encepp.eu/encepp/viewResource.htm?id=21029) | Portugal | 21-9-2017 |
| 55 | [SAIL databank](https://www.encepp.eu/encepp/viewResource.htm?id=48013) | United Kingdom | 4-7-2022 |
| 56 | [SIDIAP](https://www.encepp.eu/encepp/viewResource.htm?id=104903) | Spain | 12-5-2023 |
| 57 | [Sylvia Lawry Centre for MS Research Registry](https://www.encepp.eu/encepp/viewResource.htm?id=105030) | Germany | 24-5-2023 |
| 58 | [TREATgermany-AD (Atopic Dermatitis) Registry](https://www.encepp.eu/encepp/viewResource.htm?id=49775) | Germany | 15-11-2022 |
| 59 | [The UK FSHD Patient Registry](https://www.encepp.eu/encepp/viewResource.htm?id=37237) | United Kingdom | 17-9-2020 |
| 60 | [UK CF Registry](https://www.encepp.eu/encepp/viewResource.htm?id=36763) | United Kingdom | 14-8-2020 |
| 61 | [UK Duchenne and Becker](https://www.encepp.eu/encepp/viewResource.htm?id=48989) | Ireland | 15-9-2022 |
| 62 | [UK Myotonic Dystrophy Patient Registry](https://www.encepp.eu/encepp/viewResource.htm?id=37234) | United Kingdom | 17-9-2020 |
| 63 | [UK National Neonatal Research Database](https://www.encepp.eu/encepp/viewResource.htm?id=103457) | United Kingdom | 8-2-2023 |
| 64 | [UK Renal Registry - Renal diseases](https://www.encepp.eu/encepp/viewResource.htm?id=19236) | United Kingdom | 24-5-2017 |
| 65 | [UKHCDO - Haemophilia](https://www.encepp.eu/encepp/viewResource.htm?id=24444) | United Kingdom | 21-6-2018 |
| 66 | [UKMSR](https://www.encepp.eu/encepp/viewResource.htm?id=104249) | United Kingdom | 29-3-2023 |
| 67 | [eDRIS](https://www.encepp.eu/encepp/viewResource.htm?id=25188) | United Kingdom | 8-8-2018 |
| 68 | [epidEMcat - Multiple sclerosis](https://www.encepp.eu/encepp/viewResource.htm?id=21272) | Spain | 12-10-2017 |

Table S2. Items used as background for our assessment checklist.

| Source | Item | Item description |
| --- | --- | --- |
| EUnetHTA (2019) | Governance | An independent steering committee or a governing body and a data quality team with specified responsibilities are in place. These should include patient representation.  Registry governance should have an audited process for declarations of interest covering all financial contributions to the work. |
| EUnetHTA (2019) | Informed consent | The informed consent document should explain to potential participants:   - the nature, purpose of the registry and whether secondary analyses may be undertaken. - why they are candidates for participating in the registry. - what risks, benefits, and alternatives are associated with the participation. - what rights they have as research subjects. |
| EUnetHTA (2019) | Financing | Financial security to the end of the evidence development period should be demonstrated in the financial plan, solvency with a summary of income and expenditure for the previous 2 years is recommended. Also, funding sources are identified and the approx. proportions (%) of total sum from each funding source is indicated. |
| EUnetHTA (2019) | Protection, security and safeguards | The security controls specific for the registry should be specified. Risks should be identified and appropriate mitigation described. |
| EUnetHTA (2019) | Ethics | Consideration of research ethics requirements has been reported. If a research ethics committee approved the working procedures/ methodology of the registry, the process of obtaining approval is described. |
| AHRQ (2020) | Ethics and data protection | Evaluate the issues of protection of human subjects—including privacy, informed consent, data security, and study ethics—and address them in accordance with local, national, and international regulations.  Obtain review and approval by any required oversight committees (e.g., ethics committee, privacy committee, or institutional review board as applicable).  Identify appropriate personnel and facilities, including those for secure data storage. |
| AHRQ (2020) | Governance | Develop a clear, written plan for registry governance that specifies how registry decisions will be made and describes the roles of any external advisors.  Define the role of any external sponsor, including data access, use, and rights to review, participate or approve any publications. |
| AHRQ (2020) | Transparency | Consider if, when, and how to allow third parties access to data, if feasible, and the process for any such data access. Assure that any data transfers are accurate, only provide the requisite data, and maintain the privacy of patients, clinicians and health systems.  Plan how study results will be communicated on completion and whether the results will be made public, and if so, by whom.  Consider posting information on a public registry of patient registries (e.g., at the Registry of Patient Registries). |
| AHRQ (2020) | Change process | Establish a process for documenting any modifications to the research plan, since the main objective(s) and analytic plans may change over time as knowledge accumulates, and the plan for data collection and follow up may need to be adapted. |
